# Non-Invasive Biosensing for Healthcare Using Artificial Intelligence: A Semi-Systematic Review

**DOI:** 10.3390/bios14040183

**Published:** 2024-04-09

**Authors:** Tanvir Islam, Peter Washington

**Affiliations:** Information and Computer Sciences, University of Hawaii at Manoa, Honolulu, HI 96822, USA; tislam@hawaii.edu

**Keywords:** biosensor, deep learning, machine learning, healthcare, medical informatics, digital health

## Abstract

The rapid development of biosensing technologies together with the advent of deep learning has marked an era in healthcare and biomedical research where widespread devices like smartphones, smartwatches, and health-specific technologies have the potential to facilitate remote and accessible diagnosis, monitoring, and adaptive therapy in a naturalistic environment. This systematic review focuses on the impact of combining multiple biosensing techniques with deep learning algorithms and the application of these models to healthcare. We explore the key areas that researchers and engineers must consider when developing a deep learning model for biosensing: the data modality, the model architecture, and the real-world use case for the model. We also discuss key ongoing challenges and potential future directions for research in this field. We aim to provide useful insights for researchers who seek to use intelligent biosensing to advance precision healthcare.

## 1. Introduction

Wearable non-invasive biosensors, when combined with machine learning, can enable remote monitoring, diagnosis, and therapy for a wide range of health conditions. Wearable devices can record substantial amounts of unlabeled data from biosensors such as Electrodermal Activity (EDA), Electrocardiography (ECG), and Electroencephalography (EEG). Deep learning [1], a family of techniques well-suited for analysis of large data streams, has recently been used for making predictions with these data.

In this systematic review, we explore the synergy between non-invasive biosensors and machine learning, with a focus on deep learning in the field of healthcare and biomedical research. While our review is focused on non-invasive biosensors in particular, the insights and methods that we discuss can also be applied to other types of biosensors.

Rather than conducting one large systematic review, we organize this paper as a narrative review coupled with a series of targeted mini-reviews. Our goal is not to provide an exhaustive list of papers in the field of non-invasive intelligent biosensing for healthcare, as this research field is quite massive. Instead, we aim to provide demonstrative examples of the types of studies, innovations, and trends that have emerged in recent years.

Our semi-systematic review is structured as follows. We begin with an overview of common biosensors used in healthcare. We proceed with a review of essential deep learning architectures that are commonly applied to biosensing. We next describe specific application areas of biosensing in digital health, particularly remote patient monitoring, digital diagnosis, and adaptive digital therapy. In each of these sections, we perform a separate ‘tiny’ systematic review.

## 2. Common Deep Learning Architectures in Biosensing

**Figure 1:**
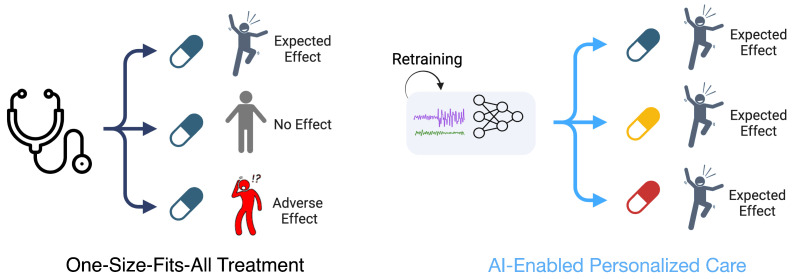
Comparison between traditional one-size-fits-all treatment strategies and personalized treatment strategies using machine learning. On the **left**, a single medication is provided to three different people, leading to three different patient outcomes. On the **right**, individualized patient data are used to personalize a treatment plan for each patient, leading to an optimized care approach with separate medications being prescribed to each patient.

Deep learning is improving the field of biosensing by enabling the analysis of the large, complex, and longitudinal data generated by biosensors. Machine learning models can enable personalization of remote treatment strategies (Figure 1). Deep learning models often achieve better performance than classical methods on biosensing-related tasks, as biosensors tend to have high sampling frequencies that result in the generation of large datasets.

In this section, we review the most common deep learning architectures used with biosensor data. Training and running deep learning models requires a significant amount of computational resources. Due to advancements in model compression, these models may now be integrated into microprocessors and run on smart phones or smart watches, allowing for real-time predictions. The goal of model compression is to create a model with significantly fewer parameters than the original model while maintaining discriminative performance. Many modern microprocessors contain hardware accelerations that are specific to deep learning, enabling close-to-real-time operations.

### 2.1. Convolutional Neural Networks (CNNs)

Around a decade ago, CNNs revolutionized the processing of images and signal data, including biosignals [2,3,4]. The CNN architecture is inspired by the natural visual perception mechanism of living organisms, performing well on data with a grid-like topology [5] such as images (2-dimensional grids of pixels) and time series or audio signals (1-dimensional grids of data). CNNs extract meaningful patterns and features from noisy and complex biological data. 1-dimensional CNNs (1D CNN) in particular are a valuable tool for biosensing.

### 2.2. Long Short-Term Memory Networks (LSTMs)

LSTM networks are a subclass of the recurrent neural network (RNN) family of architectures. LSTMs aim to solve the vanishing gradient problem that saliently exists for unmodified RNNs [6]. LSTMs have the ability to learn the dependencies throughout a sequence, making them excel at processing biosensor data, which are inherently sequential in nature [7,8].

### 2.3. Autoencoders

Autoencoders are used for dimensionality reduction and feature representation learning [9,10,11]. Autoencoders are made up of two fundamental components: an encoder and a decoder. The encoder compresses the data into a low-dimensional representation, while the decoder then reconstructs the data from this compressed form. Autoencoders can serve as a feature extraction tool for new biosignal data streams, enabling applications such as anomaly detection in the monitoring of symptoms or the diagnosis of diseases.

### 2.4. Transformers

Transformers have the potential to perform well on sequential data due to the use of the self-attention mechanism. This makes them suitable for health monitoring applications in the analysis of the temporal evolution of the physiological signals. The self-attention mechanism enables parallel processing of data during both training and inference.

### 2.5. Model Selection Considerations

Selecting an appropriate deep learning model for a healthcare application is a decision that requires careful consideration of several key factors [12,13,14]. The primary deciding factor is the type of data, as different architectures excel at processing differing types of data modalities; certain models are better equipped to capture intricate temporal dependencies, spatial correlations, or anomalies. Specifically, LSTMs are useful for continuous monitoring because of their ability to process and learn from sequential data, making them well-suited for analyzing physiological time-series data. Autoencoders excel in the efficient identification of anomalies and patterns in complex datasets due to their ability to encode data into a compact yet meaningful representation space. Transformers offer advantages in handling large sequences of data due to their self-attention mechanism, enabling the detection of subtle and complex patterns when trained using large datasets. One-dimensional CNNs have also demonstrated utility in making predictions from time-series data. CNNs possess the ability to effectively capture local and global patterns in sequential data.

### 2.6. Commercial Use of Deep Learning Models for Biosensing

Many products on the market use deep learning for biosensing. For instance, popular products like Fitbit and Apple provide features for tracking heart rate and activity levels using techniques that incorporate deep learning into the process [15,16]. SpeechVive leverages deep learning algorithms to analyze speech patterns, a voice-based biosignal, to offer aid to individuals living with Parkinson’s disease. The Samsung Watch uses an autoencoder and LSTM for a smart alarm system during sleep [17]. The Samsung Smartwatch uses a CNN for early detection and burden estimation of atrial fibrillation [18]. The Oura ring tracks cardiovascular activity using deep learning [19]. Oura users can follow their autonomic nervous system responses to their daily behavior based on nightly changes in heart rate. Many other wearable device companies, while not publicly releasing the details of their proprietary algorithms, most likely use deep learning.

## 3. Sensor Modalities and Corresponding Health Applications

**Figure 2:**
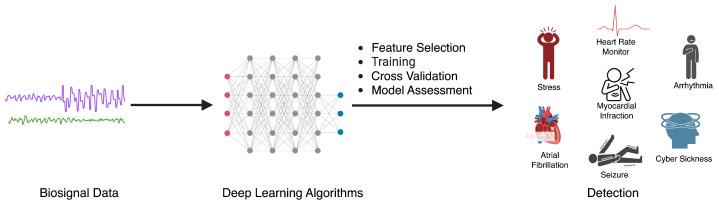
The process of utilizing deep learning algorithms for the detection of various health conditions using biosignal data, where physiological signals are fed as input to a deep learning model that makes a prediction of a health event of interest.

Here, we describe common sensor modalities used for biosensing and the corresponding health applications that are common to each modality (Figure 2). We note that the field of biosensing is so vast that we do not provide a comprehensive overview of every biosensor that has ever been used for predictive modeling. Instead, we select 3 common yet complementary modalities as demonstrative examples.

### 3.1. Deep Learning with EEG

Prior work has successfully used EEG signals to classify emotions [20], detect seizures [21], and measure sleep [22]. EEG is a widely used biosignal for the study of cognitive functions, including perception, memory, focus, language, and emotional processing.

Using Web of Science and PubMed, our search query focused on articles from 2020 to 2023 that addressed EEG-based classification and detection, with titles containing *classification* or *detection* and abstracts mentioning *CNN* and *EEG*. This approach was also used for LSTMs, Autoencoders, and Transformers. We excluded review articles, pilot studies, and duplicates from our search. There was some overlap between the Web of Science and PubMed results. Table 1 summarizes these papers.

### 3.2. Deep Learning with EDA

EDA is a common indicator in affective computing due to its sensitivity to physiological changes. However, the challenge of individual variability in EDA responses necessitates advanced analytical approaches to effectively interpret and utilize these signals. EDA is sensitive to skin hydration levels [80], making EDA a pivotal tool for capturing physiological arousal and emotional responses [81].

Our search in the Web of Science and PubMed focused on deep learning models using EDA, and we searched for papers published between 2020 and 2023. We also applied the research area filter to be Computer Science and Medical Informatics. There was some overlap between the Web of Science and PubMed search results. We further filtered papers based on their relevance to intelligent biosensing. Table 2 summarizes the selected papers.

### 3.3. Deep Learning with ECG

Cardiovascular disease is a leading cause of death globally. Factors such as stress and psychological distress have been linked to an increased risk of cardiovascular disease, especially in younger people [104]. ECGs are sensors measuring the electrical activity of the heart. ECGs are used for the diagnosis of heart problems, such as arrhythmias and heart attacks [105]. However, ECGs produce a large amount of data that are hard to scrutinize manually [106]. Deep learning algorithms can be trained to identify patterns in ECG data that are associated with different heart conditions [107]. This can help doctors to diagnose heart problems more efficiently and scalably.

Using Web of Science and PubMed, we performed a targeted search for recent (2020–2023) research using deep learning to analyze ECG data for classification and detection tasks. Specifically querying CNN, LSTM, Autoencoder, and Transformer architectures, we screened for *classification* or *detection* in titles and model references in abstracts. After assessing their relevance to computer science and cardiology, we identified a final set of papers. There was some overlap between the Web of Science and PubMed search results. Table 3 provides a summary of the identified papers.

### 3.4. Consideration for Selecting Biosensors for a Digital Health Application

Each type of biosensor, mentioned above, is often useful for a small range of health conditions. EEG is used for measuring electrical activity of the brain, making it particularly suitable for neurology and cognitive science research. EDA, on the other hand, is less complicated to measure from an end-user perspective than EEG and can be used for applications such as psychological research, stress monitoring, and affective computing. ECG, measuring the electrical activity of the heart, is used in cardiology. We notably did not review multimodal models. However, it is worth noting that it is likely that combining multiple biosensors into a single predictive model might enable the detection of previously unexplored health conditions and events that are infeasible to predict using a single modality alone.

## 4. Digital Health Applications using Biosensors

Machine learning-based biosensing can be applied to several areas in healthcare. Here, we summarize recent literature in three common applications of biosensing: remote patient monitoring, digital diagnostics, and adaptive digital interventions. We distinguish between remote patient monitoring and digital diagnosis in the following manner: a diagnosis is made of a disease that the patient may or may not have, while remote monitoring focuses on *symptoms* that can periodically occur for the patient regardless of whether the patient has a disease diagnosis.

### 4.1. Remote Patient Monitoring

Biosensing technology, when combined with machine learning, opens up new prospects for improving remote patient care through continuous physiological data analysis outside of the clinic. Continuous physiological data analysis provides opportunities to significantly improve patient care by providing the clinician with more detailed status reports about each patient [166].

Key technical challenges for remote patient monitoring include efficiently processing and transmitting complex physiological data to optimize energy use and sustainability [167,168], accurately interpreting multifaceted biosensing signals [169], and creating adaptive systems that provide robust monitoring across a diverse array of patients [170,171]. Additional human-centered challenges involve the need to respond quickly enough to develop supportive technologies amidst the detection of an adverse health event [172].

We performed a search on Web of Science using the search terms *remote patient monitoring*, *telehealth*, *wireless health monitoring*, and *machine learning* along with the use of *biosensors* mentioned in the abstracts. Table 4 displays the application and overview of these remote patient monitoring papers, focusing on the application rather than the technique or evaluation.

### 4.2. Digital Diagnostics

There are multiple key challenges that must be addressed before translation of biosensor- based digital diagnostics into clinical settings. First, the heterogeneity of data can complicate biosensing-based diagnostics. Intrinsic variability common in biosensors [176,177] and the necessity for sensor stability plus consistent performance throughout a wide range of environmental conditions create a significant challenge for machine learning modeling. Second, the models must be robust enough to handle and interpret the vast, complex datasets that biosensors generate without overlooking nuances that can occur at relatively small time-scales. Finally, biosensors used for nuanced and possibly even subjective diagnoses require high analytical sensitivity and specificity to interpret signals that differ subtly between people [178].

We performed a search for digital diagnostics in the Web of Science for papers with a title matching *biosensor*, *healthcare*, and *ML* coupled with the keywords *classification* or *detection*, focusing on the last 5 years. We further filtered papers that were not relevant to diagnostics. We list the identified papers in Table 5, focusing on the application rather than the technique or evaluation.

### 4.3. Adaptive Digital Interventions

Digital health interventions powered by machine learning have the potential to improve health outcomes through therapeutics that are delivered in a just-in-time manner [185]. However, these interventions pose critical challenges, such as data privacy. Though these technologies hold great promise for providing the basis for real-time intervention for a wide spectrum of health conditions, their use must be fine-tuned and validated so that proper, reliable responses may be used in actual practice [186,187,188].

We performed a search on Web of Science for health interventions, using the terms *Randomized Controlled Trials*, *wearable sensor*, and *biosensor*, along with *machine learning* in the abstract. We excluded review articles and protocols. Table 6 outlines the clinical endpoint, the application effectiveness metrics, and a summary of the study, offering insights into the impact and scope of digital health interventions using intelligent biosensing.

### 4.4. Considerations for Integrating Wearable Sensors in Digital Health Systems

The integration of wearable sensors in digital health systems requires careful consideration of human factors such as interoperability, interpretability, bias, privacy, and user compliance.

Interpretability is necessary to enable clinicians to understand the reasoning behind a models’ decision making [196]. However, deep learning models are inherently not interpretable, requiring black-box solutions that only provide partial explainability.

Biases in machine learning models can be harmful to biomedical research because incorrect predictions made by digital health technologies may disproportionately affect some demographic groups. Bias may exist at several phases of the model development cycle, including data collection, model optimization, and model calibration [197].

There is always a risk of privacy violations when dealing with sensitive health data for patient monitoring or disease diagnosis [198,199]. A possible solution is federated learning, where a separate model is trained on each individual’s device. This solution integrates well with the idea of model personalization.

Finally, addressing wearability and other user-centered design issues is needed to improve patient compliance. It is well documented that users often do not properly use their wearables. Several machine learning models have been considered for addressing these adherence issues [200,201,202].

## 5. Challenges and Opportunities

The integration of deep learning with biosensor data presents several challenges and corresponding opportunities for advancing healthcare. We discuss these here.

### 5.1. Challenges

There are several technical challenges that can hinder the development and deployment of intelligent biosensing. We discuss three key challenges: (1) variability across human subjects, (2) noise, and (3) the necessity of complex and highly parameterized models to make sense of biosignal data streams.

Variability across human subjects is a major challenge for model generalization [82,84]. This can compromise the ability to generalize a model to new subjects [25,68,72], leading to inconsistent signals being recorded between people. Individual physiological characteristics can have a major impact on the patterns that are present within the data, reducing the accuracy of models when applied to new participants.

Another key challenge is the inherent noisiness of real-world biosignal data. This noise can arise both from the underlying biosensor data as well as from irregular labeling practices. The subjectivity and difficulty of forecasting outcomes, such as stress levels, may add to this noise. These issues have been shown to negatively impact model performance [86,88,103].

Model complexity presents yet another core challenge, leading to difficulties in the interpretation of results [25,68]. Deep learning models are powerful but can be too complex to understand easily. This ‘black box’ nature of deep learning models can render it difficult for doctors to trust and use these models.

### 5.2. Opportunities

The challenges described above can be solved by a number of promising techniques that have recently emerged in the literature. We describe two key research opportunities for the field: (1) personalization using self-supervised learning and (2) multimodal explainable artificial intelligence.

There is an opportunity for the research community to innovate in the personalization of deep learning models using biosensor data. Personalizing models to account for variability among subjects has the potential to increase model performance, especially when coupled with multimodal learning approaches [86,88,203,204,205,206,207]. Such personalization can be enhanced with self-supervised learning, where a model is pre-trained on the massive unlabeled data streams that are generated when a user passively wears a device with one or more biosensors.

There is also an opportunity to combine innovations in multimodal deep learning with explainable artificial intelligence. Multimodal explainable machine learning approaches designed for time-series data have the potential to improve the clinician and patient’s understanding of automated or semi-automated decision making processes.

## 6. Conclusions

We have reviewed common deep learning architectures, sensor modalities, and healthcare applications in the field of intelligent biosensing. The fusion of biosensing and artificial intelligence can lead to improved precision healthcare via scalable and accessible remote monitoring platforms, digital diagnostics, and adaptive just-in-time digital interventions.

## Figures and Tables

**Table 1:** Overview of deep learning algorithms used in different EEG-based studies. In some studies, more than one dataset is used. They are separated by a “/”.

Neural Networks	Application	Ref. No.	Dataset Sample Size	Performance
CNN	motor imagery classification	[23]	9 [24]	accuracy: 93.74%
	seizure classification	[25]	352 [26]	accuracy: 88.30%
	Alzheimer’s and mild cognitive impairment classification	[27]	90	accuracy: 92%
	mental state classification	[28]	12/15 [29]	accuracy from [28]: 86.29% and accuracy from [29]: 93.68%
	mild cognitive impairment classification	[30]	36	accuracy, sensitivity, specificity, and AUC all above 99%
	Alzheimer’s classification	[31]	48	accuracy: 93.04% and F1-score: 93.09%
	depression classification	[32]	92	accuracy: 98.95% and F1-score: 98.46%
	sleep stage classification	[33]	100 [34]	accuracy: 99.39%
	quiet neonatal sleep classification	[35]	19 [34]	accuracy: 94.07%
	error-related potential (ErrP) classification	[36]	6	accuracy: 86.46%
	sleep spindle classification	[37]	141	sensitivity: 91.9% to 96.5% and specificity: 95.3% to 96.7%
	sleep apnea classification	[38]	2650 [39]	accuracy: 69.9%
	Parkinson’s classification	[40]	15 [41]	AUC: 0.99
LSTM	eye-blink and muscular artifact classification	[42]	40 [43]	accuracy: 97.4%
	sleep stage classification	[44]	40 [45]	PCC for 4-class classification: 90.80% and 2-class classification: 83.56%
	emotion quantification from facial expressions	[46]	28 [47]	RMSE: 0.053±0.029
	neurodegenerative disease classification	[48]	68	accuracy: 75.3%
	ischemic stroke subtype classification	[49]	2310	AUC: 0.774
	early mild cognitive impairment classification	[50]	27 [51]	accuracy: 96.41%, sensitivity: 96.55%, and specificity: 95.95%
	epileptic seizure detection	[52]	10	accuracy for three-class and four-class: 95%, accuracy for five-class: 93.5%
	mental task classification	[53]	32	F1-score: 85%
	driver fatigue detection	[54]	23	accuracy: 87.3%
	focal and feneralized epilepsy detection	[55]	50	accuracy: 96.1%
	cyclic alternating pattern (CAP) classification	[56]	16	ROC: 0.82, accuracy: ranging from 77% to 79%
	stress classification	[57]	40	accuracy: 93.17%
Autoencoder	epileptic seizure detection	[58]	5 [26]	2-Class classification accuracy: 99.53% and multi-class classification accuracy: 98.67%
	motor imagery classification	[59]	9 [60]/14 [61]	accuracy from first dataset: 75.7% and accuracy from second dataset: 95.4%
	epileptic seizure detection	[62]	5 [63]/21 [64]	accuracy from [63]: 75.7% and accuracy from [64]: 95.4%
	Alzheimer’s classification	[27]	90	accuracy: 89%
	sleep apnea classification	[65]	994 [66]	accuracy: 90.26%
	epilepsy classification	[67]	23	accuracy: 99.08±0.54%
Transformer	Alzheimer’s classification	[68]	88 [69]	accuracy: 83.28%
	seizure detection	[70]	22 [71]	accuracy: 96.31% and F1-score: 96.32%
	steady-state visual evoked potential (SSVEP) classification	[72]	10 [73]/35 [74]	accuracy from [73]: 88.37% and accuracy from [74]: 83.19%
	epilepsy detection	[75]	121 [76]	accuracy: 85%
	sleep stage classification	[77]	21 [78]	accuracy: 90.26% and F1-score: 86.51%
	seizure detection	[79]	23	accuracy: 96.15%

**Table 2:** Overview of deep learning algorithms used in different EDA-based studies. In some studies, more than one dataset is used. They are separated by a “/”.

Neural Networks	Application	Ref. No.	Dataset Sample Size	Performance
CNN	emotion classification	[82]	32 [83]	F1-score for valence and arousal: 71.41% and 79.3%, respectively
	acute pain classification	[84]	38 [85]	accuracy: 91.3%
	stress detection	[86]	15 [87]	RMSE: less than 0.05
	stress detection	[88]	15 [89]	accuracy: 79.65% and F1-Score: 75.22%
	heat-induced pain classification	[90]	10	accuracy: 75.57%
LSTM	acute pain classification	[84]	38 [85]	accuracy: 95.2% and F1-score: 74.9%
	cybersickness classification	[91]	9 [92]	accuracy: 96.85%
	pain intensity classification	[93]	29 [94]	F1-score: 81% and AUC: 0.93
	skin hydration level classification	[95]	16 [96]	accuracy: 97.83%
Autoencoder	stress detection	[97]	58 [98]/	
			62 [99]/	accuracies for [98,99,100,101]: 97.4%, 96.5%, 88% and 84.8%, respectively;
			22 [100]/	F1-scores for [98,99,100,101]: 97%, 95%, 87% and 85%, respectively
			48 [101]	
	epileptic seizure detection	[102]	166	sensitivity: 83.9% and false positive rate: 35.3%
Transformer	stress detection	[103]	14 [103]	accuracy for 2-class task: 93.28%, 3-class task: 88.75%, and 4-class task: 84.85%

**Table 3:** Overview of deep learning algorithms used in different ECG-based studies. In some studies, more than one dataset is used. They are separated by a “/”.

Neural Networks	Application	Ref. No.	Dataset Sample Size	Performance
CNN	arrhythmia detection	[108]	47 [45,109]	1D CNN accuracy: 90.93% and 2D CNN accuracy: 99%
	multi-class arrhythmia detection	[110]	6877 [110]	F1-score: 81.2%
	myocardial infarction detection	[111]	290 [45,109]	accuracy: 98.5%
	myocardial infarction detection	[112]	11 [45,109]	accuracy with noise: 93.53% and without noise: 95.22%
	atrial fibrillation detection	[113]	89 [114]	specificity: 98.96% and sensitivity: 86.04%
	heart abnormality classification	[115]	480	accuracy: 99.98%
	short-term atrial fibrillation detection	[116]	25 [117]	F1-score: 88.18%
	automatic arousal detection	[118]	6600 [119]	AUC: 0.86
	acute coronary syndrome-related disease classification	[120]	-	accuracy: 71%
	sleep classification	[121]	136	accuracy: 86.3%
	ADHD and CD classification	[122]	123	accuracy: 96.04%
	stress detection	[123]	-	accuracy: 88.4%
	shockable arrhythmia classification	[124]	18	AUC: 0.995
	driver arrhythmia classification	[125]	-	accuracy: 88.99%
	cardiovascular disease classification	[126]	10,646	accuracy: 95.08%
	arrhythmia detection	[127]	47 [117]/290 [128]	accuracy for [117]: 98.66% and accuracy for [128]: 95.79%
	inter-patient ECG classification and arrhythmia detection	[129]	47 [117]	accuracy: 98.18%
	cardiac rhythm classification	[130]	1928 [131]	F1-score: 89%
	sleep apnea detection	[132]	70 [133]	per-recording accuracy: 100% and per-minute accuracy: 85.8%
LSTM	arrhythmia classification	[134]	47 [45,109]	accuracy: 99%
	ECG signal classification	[135]	47 [45,109]	accuracy: 99.39%
	heart failure classification	[136]	40,000 [137]	accuracy: 99.09%
	real-time anomaly detection and classification of 1D ECG signals	[138]	162	accuracy: 100%
	premature ventricular contraction	[139]	47 [109]	accuracy: 98.5%
	atrial fibrillation detection	[140]	47 [109]	accuracy: 93.05%
Autoencoder	cardiac arrhythmia classification	[141]	47 [45,109]	accuracy for VEB: 94.9% and accuracy for SVEB: 94.4%
	QRS detection	[142]	47 [45,109]	accuracy: 99.6%
	detection and localization of myocardial infarction	[143]	52 [45]/148 [144]	MI detection accuracy: 99.87% and MI localization accuracy: >99%
	ECG beat classification	[145]	47 [45,109]	OAA-MLP accuracy: 99.32% and OAO-MLP accuracy: 99.14%
	anomaly detection	[146]	47 [45,109]	F1-score: 93%
	ECG heartbeat classification	[147]	47 [148]	accuracy: 99.99%
	atrial fibrillation classification	[149]	25 [45]	accuracy: 99.25%
	beat-by-beat atrial fibrillation detection	[150]	12,186 [151]/25 [109]	F1-score for [151]: 88% and F1-score for [109]: 87% and
	heart abnormalities detection	[152]	105 [153]	accuracy: 98.59%
Transformer	arrhythmia classification	[154]	6877 [155]	F1-Score: 78.6%
	ECG heartbeat classification	[156]	10 [157]	accuracy: 99.32%
	arrhythmia detection	[158]	47/25 [45,109]	4-categories accuracy: 99.12%, 8-categories accuracy: 99.49%, and binary classification accuracy: 99.23%
	heartbeat arrhythmia classification	[159]	337	accuracy: 98%
	ECG classification	[160]	110 [155]	accuracy: 86% and F1-score: 83%
	stress detection	[161]	15 [87]	F1-score: 97%
	classification of tetanus severity	[162]	110 [163]	F1-score: 88%
	inter-patient congestive heart failure detection	[164]	18 [45]/15 [165]	accuracy: 98.88%

**Table 4:** Summary of remote patient monitoring applications at the intersection of biosensing and machine learning.

Reference No.	Application
[166]	continuous remote patient monitoring in heart failure management
[167]	remote patient monitoring optimization in IoMT networks
[168]	energy-efficient patient monitoring in IoHT networks
[169]	ambient intelligent system for psychiatric emergencies
[170]	stroke volume monitoring in congenital heart disease via wearable technology
[172]	COVID-19 decompensation detection via wearable biosensors
[173]	evaluating remote patient monitoring and education technology for COVID-19 symptoms
[174]	IoT-Aware smart hospital system for patient and asset monitoring
[175]	remote human vital signs monitoring with a 77 GHz FMCW radar

**Table 5:** Summary of digital diagnostics using biosensing and machine learning.

Reference No.	Application
[179]	early detection of acute myocardial infarction
[178]	real-time early detection of cadaverine for periodontal disease diagnostics and personalized treatment plans
[176]	simultaneous detection of protein biomarkers in urine in point-of-care settings
[177]	heart failure diagnosis with electrochemical sensor from biomarkers in saliva
[180]	detection of BNP biomarkers in serum
[181]	alternative to PCR tests to detect coronaviruses, including MERS-CoV and SARS-CoV-2
[182]	detection of SARS-CoV-2 and influenza using liquid-gated graphene field-effect transistors
[183]	public health surveillance through pathogen detection in water
[184]	detection of serum amyloid A (SAA) and C-reactive protein (CRP) biomarkers

**Table 6:** Summary of digital health intervention studies, including clinical endpoints, metrics indicating the effectiveness of the intervention, and a summary of the findings from the study.

Ref. No.	Clinical Endpoint	Application Effectiveness	Summary
[189]	Moderate to Vigorous PA (MVPA)	4.3 min/week increase vs. controls	The intervention using a wearable device (Fitbit One) and SMS prompts showed a short-term increase in physical activity among overweight and obese adults, but the effect was not sustained beyond the first week of the 6-week study period.
[190]	Atrial Fibrillation Detection Rate	9.4% enrollment increase with optimized campaign vs. baseline	The mSToPS trial focused on screening for undiagnosed atrial fibrillation (AF) using a wearable ECG sensor patch, targeting individuals at increased risk. The study emphasized the importance of early detection of AF, a significant contributor to stroke and mortality, to potentially initiate preventative treatment and reduce health risks.
[191]	Heart Rate (Awake/Asleep)	Detected clenbuterol effect from Day 3 (Awake, +8.79 bpm, p=0.001) and Night 1 (Asleep, +3.79 bpm, p=0.04)	This study successfully demonstrated the potential of using smartwatch-based heart rate monitoring to detect clenbuterol-induced changes in heart rate during clinical trials, proving particularly effective and sensitive while participants were asleep.
[192]	Global Perceived Effect (GPE)	Higher in VRRS (4.76±0.43) vs. Control (3.96±0.65); *p* < 0.001	The study demonstrated that early virtual-reality-based home rehabilitation (VRRS) after Total Hip Arthroplasty was as effective as traditional rehabilitation in improving functional outcomes, with participants in the VRRS group expressing higher satisfaction with their rehabilitation program. This indicates that VRRS can be a viable and patient-preferred alternative to conventional methods, warranting further exploration.
[193]	Influenza Prediction Accuracy	81% accuracy 2 days before major symptoms	The study demonstrated employing wearable technology for continuous monitoring of physiological parameters for early flu detection and surveillance, offering insights into the natural progression of the disease and facilitating timely healthcare interventions during outbreaks.
[194]	System Compliance	Median 11.57 days of use out of 14-day period (87% completion rate)	This study validated the mHealth system’s ability to passively and unobtrusively monitor and evaluate Parkinson’s disease symptoms, including an evaluation algorithm, indicating its potential to enhance disease management and patient care in real-life settings. Future research is needed to confirm these benefits and to further explore the system’s impact on disease management.
[195]	Medication Adherence	30% increase in confirmed daily doses with WOT (93% with WOT vs. 63% with DOT)	The study demonstrated that Wirelessly Observed Therapy (WOT), a digital patient self-management system involving an edible ingestion sensor, wearable patch, and mobile device, accurately detected medication ingestions and confirmed daily adherence to tuberculosis (TB) treatment more effectively than Directly Observed Therapy (DOT).
